# Optimization of Topography and Surface Properties of Polyacrylonitrile-Based Electrospun Scaffolds via Nonoclay Concentrations and its Effect on Osteogenic Differentiation of Human Mesenchymal Stem Cells

**DOI:** 10.22037/ijpr.2021.115119.15208

**Published:** 2021

**Authors:** Fatemeh Sadat Tabatabaei Mirakabad, Simzar Hosseinzadeh, Hojjat Allah Abbaszadeh, Vahideh Zeighamian, Maryam Sadat Khoramgah, Hossein Ghanbarian, Javad Ranjbari, Bahram Kazemi

**Affiliations:** a *Cellular and Molecular Biology Research Center, Shahid Beheshti University of Medical Sciences, Tehran, Iran. *; b *Department of Medical Biotechnology, School of Advanced Technologies in Medicine, Shahid Beheshti University of Medical Science, Tehran, Iran. *; c *Department of Tissue Engineering and Regenerative Medicine, School of Advanced Technologies in Medicine, Shahid Beheshti University of Medical Sciences, Tehran, Iran. *; d *Laser Application in Medical Sciences Research Center, Shaid Beheshti University of Medical Sciences, Tehran, Iran. *; e *Department of Medical Biotechnology, Faculty of Advanced Medical Sciences, Tabriz University of Medical Sciences, Tabriz, Iran. *; f * Hearing Disorders Research Center, Loghman Hakim Hospital, Shahid Beheshti University of Medical Sciences, Tehran, Iran. *; g *Department of Biology and Anatomical Sciences, Faculty of Medicine, Shahid Beheshti University of Medical Sciences, Tehran, Iran.*

**Keywords:** Nanoclay, Polyacrylonitrile (PAN), Topography, Osteogenic Differentiation, Mesenchymal Stem Cell (MSCs)

## Abstract

Nowadays, mesenchymal stem cells (MSCs) are the most widely used cell sources for bone regenerative medicine. Electrospun polyacrylonitrile (PAN)-based scaffolds play an important role in bone tissue engineering due to their good mechanical properties, which could be enhanced by the presence of nanoparticles such as nanoclay. This study evaluated the *in-vitro* effect of different concentrations of nanoclay in surface characteristic properties of PAN-based electrospun nanofiber scaffolds and the osteogenic differentiation ability of adipose-derived mesenchymal stem cells (AD-MSCs). After electrospinning nanofibers, their structure were assessed through some characterization tests. Then AD-MSCs isolation and characterization were done, and the cell attachment and the biocompatibility were determined. Finally, osteogenic differentiation-related markers, genes, and proteins were studied. Clay-PAN25% electrospun nanofiber scaffold could support attachment, proliferation, and osteogenic differentiation of AD-MSCs better than other groups. Also, nanoclay could enhance the properties of PAN-based scaffolds, such as fiber diameter, topography, surface charge, hydrophilicity, roughness, and degradation, as well as osteogenic differentiation of cells. As a result, Clay-PAN25% with the highest concentration of nanoclay was found as a promising biodegradable and cost-effective scaffold for osteogenic differentiation of AD-MSCs.

## Introduction

Due to the inability of clinical procedures to heal bone defects, tissue engineering (TE) has emerged as a promising technology nowadays. The first element which is required for TE is the cell source. Mesenchymal stem cells (MSCs) are primary cellular sources widely used for cell therapy programs (1). Also, scaffolds are needed as the second element that can mimic an environment similar to the Extra Cellular Matrix(ECM) (2). Scaffolds, as a physical supporter, play an important role by providing three-dimensional(3D) substrates for cell physiological behaviors (3). So, designing a scaffold that mimics the 3D structure with properties of natural tissues is challengeable (4). Natural and synthetic polymers have been popular for bone TE due to their diverse properties and bioactivity (5). Also, nanofiber scaffolds have a good resemblance to the physiological environment due to their high surface-to-volume ratios, porosity, and significant mechanical properties (6). Electrospinning as a versatile, cost-efficient, flexible and powerful technique was applied to prepare nanofibers (7). Polyacrylonitrile (PAN) electrospun synthetic polymer is one of the most common materials due to its appropriate physicochemical properties (8). Moreover, nanomaterial modified scaffolds prepare some surface topographical changes and finally affect on cell behaviors (9). Clay nanoparticles/nanoclay are mineral layered silicate constructions (10), which due to their simplicity of construction, biocompatibility, and cost-effectiveness, have a great potential for applications in TE (11) to improve the properties of polymeric scaffolds (12). The silicate base and the surface reactivity of nanoclay (13) interact with scaffold materials, ECM, and intracellular signaling pathways (10), leading to stimulation and differentiation of cells. Consequently, the combination of polyacrylonitrile scaffold with different percentages of nanoclay will be considered as a suitable substrate for osteogenic differentiation of MSCs. Therefore, in the present study, different concentrations of nanoclay were used in PAN-based scaffold via electrospinning technique, and their impacts on MSCs osteogenic differentiation, topography, and surface properties of scaffolds were evaluated. 

## Experimental

*Fabrication of Clay-PAN and PAN Electrospun Scaffolds*

In this study, a range of electrospun scaffolds was fabricated through varying the nanoclay concentrations within the PAN scaffolds. Three kinds of Clay-PAN scaffold (15%, 20%, and 25%) were synthesized with 0.15, 0.20, and 0.25 g of montmorillonite clay powder (Sigma-Aldrich, USA) which were dissolved in 4 mL of DMF (Sigma-Aldrich, USA) separately and sonicated for 20 min in the warm water bath of 60 °C. Then 0.28 g of PAN (Poly acrylonitrile) polymer powder (Aldrich 25014-41-9, Mw: 150,000 Da) was added to each solution and stirred at 700RPM in RT for 4hours. Also, PAN scaffold fabrication was done with 0.28 g of PAN powder which dissolved in 4 mL of DMF and stirred 4 h at 700RPM in RT. Then, the separated solutions were run in an electrospinning device (Nano Spinner, Iran) at a voltage of 15 kV throughout, and space between the nozzle tip and collector was set up at 15 cm. So, the solvent evaporated, and a collector collected the scaffold fibers with a rotating drum speed of 400 rpm with the flow rate of 0.3 mL/h. 

*Characterization of Electrospun Nanofibers*

Morphology and structure of scaffolds were evaluated by scanning electron microscope (SEM). Gold layer was coated on the surface of scaffolds, then they were observed via Philips/FEI XL30FESEM (Philips, Eindhoven, Nederland). 

Nanoclay dispersion were analyzed by using Transmission electron microscopy (TEM). The nanoclay particles were dispersed in ethanol and deposited on the carbon film copper network, and the Clay-PAN25% electrospun nanofibers were directly deposited on the copper network. Then they were observed by TEM (JEM-2100F, JEOL, Japan) at the voltage of 150 kV. 

Atomic force microscopy (AFM) topography information was acquired using NanowizardII by JPK Instruments (Berlin, Germany) in tapping mode, to measure the nanofibers surface roughness. Four points on fiber surface were selected randomly, and the roughness was measured (14, 15).

To recognize structures, molecular components and functional groups of scaffolds, the Fourier Transform Infrared Spectroscopy (FTIR) were done by ALPHA-FTIR Spectrometer (Bruker) with the wavenumber range of 4000–400 cm^- 1^. 

The surface hydrophilicity of scaffolds was measured by contact angle. The scaffolds were cut and fixed on microscope slides then 2 μL of deionized water was placed on the surfaces at RT, and contact angles was assessed via a 15 plus OCA instrument (Data Physics, Germany) in less than 1 min. Final drop shaped images were captured with charge-coupled device camera and analyzed by software. 

The mechanical properties of nanofibers were evaluated by tensile test. The scaffolds were evaluated through a mechanical testing machine (Santam (Iran, SPM20)) for stress-strain response at a 10 mm min^−1^ crosshead speed. A digital micrometer measured the thickness of rectangular shapes of mats, and when the samples were load in 0.5 kN, the typical stress–strain response of scaffolds was considered as a stress–strain curve (16). 

The zeta potential which is related to the surface charge, was assessed through a Malvern Zetasizer Nano ZS (Malvern Instruments, Ltd.; Worcestershire, UK). One milligram per milliliter concentrations of all samples were prepared in PBS (Phosphate buffer, Non-saline, pH 7.4, Sigma, USA) and were analyzed (17). 

The biodegradation behaviors of scaffolds were studied in PBS (Sigma, USA) at 37 °C. The scaffolds (7 mm × 5 mm) were weighed (W_0_), and followed by immersion in PBS at 37 °C for 60 days. After the incubation period at the selected time points, three samples of each scaffold were removed and rinsed with deionized distilled water, then dried in a vacuum oven at 60 °C for 12 h and weighed (W_t_). The weight loss (%) was determined using the following formula: Weight loss (%) = (W_0_−W_t_)/W_0 _× 100%, where W_0_ is the starting dry weight and W_t_ is the dry sample weight after removal. 

*Isolation and Characterization of AD-MSCs*

After the liposuction surgical procedure in Taleghani Hospital Tehran, MSCs were isolated from human subcutaneous adipose tissues through informed consent signed by the donor. Isolated tissues were kept in a Hanks Buffer Salt Solution (HBSS) container with streptomycin and penicillin. Isolated tissues were cut after washing by PBS. The procedure of MSCs isolation was carried out using an enzymatic procedure through incubation in DMEM (Gibco, Germany) containing 0.2% collagenase type IA (Sigma, USA) for 40 min at 37 °C. Then centrifuged at 1500 rpm for 10min and cells were suspended in DMEM with 15% fetal bovine serum FBS (Gibco, Germany) and 1% penicillin and streptomycin (Gibco, Thermo Fisher, USA) in the tissue culture flasks and incubated at 37 °C humidified with 5% CO2 atmosphere to reach proper density. In the current study, AD-MSCs in passage two were used for *in-vitro* tests. AD-MSCs were characterized by evaluation of MSCs surface markers via flow cytometric analysis, in which fluorescent isothiocyanate (FITC)-conjugated mouse anti-human CD44, CD73, CD90, CD105, CD45, and CD34 (Sigma- Aldrich, USA) were applied (18).

*Cytocompatibility and Viability Study*

AD-MSCs were cultured in DMEM with 10% Fetal Bovine Serum (FBS) (Gibco, Germany) and 1% streptomycin/penicillin. The cells were washed by PBS and detached by 0.25% trypsin-EDTA solution (Gibco, Germany). 1 × 10^4^ cells/wells were seeded on scaffolds of 96-well. MTT assay was done on days 1, 3, 7, 14 and 21 via 3- [4,5dimethylthiazol- 2yl]-2,5-diphenyl tetrazolium bromide (MTT) (Sigma- Aldrich, UK). After the desired time, the scaffolds were adjacent to the MTT solution for 3 h in the incubator. Then dimethyl sulfoxide (Sigma-Aldrich, UK) was added. Finally, the optical density was determined through the Elisa reader instrument (BioTekEL×800) at 570 nm wavelengths. All samples were triplicates, and TCPs were considered as control groups.

*Human Mesenchymal Stem Cell Adhesion Studies*

The attachment of cells was approved through SEM images and the 4,6-Diamidino2-phenylindole staining test. SEM images were taken after 21 days. First, the samples were washed with PBS. Then they were fixed by glutaraldehyde 4.5% for 2 h. Second, the scaffolds were dipped in 60–100% ethanol overnight and dried. Finally, the surface of the scaffolds was covered by a thin layer of gold, and the images were obtained by Hitachi SEM (SU3500, Japan).

Also, cell attachment was approved by the DAPI staining test. 1 × 10^4^ cell/well were seeded on the scaffolds, and DAPI staining were done after 14 and 21 days. Then, they were washed and incubated with paraformaldehyde 4% for 10 min and washed. Formerly, TritonX-100 (0.1%) was applied for 2min, and the scaffolds were washed. To stain the cells’ nuclei, DAPI stain (Sigma-Aldrich, UK) were used in the dark place for 5min. They were washed with PBS and before taking photographs by Nikon fluorescent microscope (Eclipse Terminal Emulator 2000-S, Japan), they were preserved in dark and cold places (19). 

*Osteogenic Differentiation Markers Studies*

Trichrome staining and alkaline phosphatase (ALP) activity, as common osteogenic markers, were applied to examine the osteogenic differentiation. For preparation, scaffolds were fixed in paraformaldehyde 4%, embedded in paraffin and each of them was divided up to 5 μm pieces used for Masson’s trichrome staining. Routine histological protocols of masson’s staining were performed step by step to quantify the collagen secretion with blue stain in the osteogenic differentiation process (20-22). The collagen percentage was estimated through a custom ImageJ macro according to a color deconvolution technique.

ALP activity was measured by the total protein of MSCs extracted by RIPA lysis buffer at days 7, 14, and 21. The achieved lysate was centrifuged (15000RPM, 4 °C, 15 min), and p-nitrophenyl phosphate (pNPP) was applied as a phosphatase substrate (ALP Kit, Pars Azmoon Iran) which determined the ALP activities of the supernatant at 450 nm. The acquired ALP enzyme activity level was normalized against total protein. In this study, PAN scaffold and TCPs were considered as control groups.

*Primer Design, RNA Extraction, and cDNA Synthesis*

Oligo7 software was applied to design specific primers for osteogenic differentiation genes such as runt-related transcription factor2 (RUNX2), collagen type I alpha (Colα), osteocalcin (OCN) and osteonectin (ON) as well as GAPDH in place of the housekeeping gene. The sequences of primers are demonstrated in Table1. A Hybrid-RTM kit (Gene All, Korea) was used to extract mRNAs from all samples based on the manufacturer’s manual. 1 microgram RNA, 1 μL of random hexamer primer (10 pm), 0.5 μL dNTPs mix (10 mM), 2 μL reveres transcription buffer, and 0.3 μL *Reverse Transcriptase* enzyme (200 U/ μL) (Yekta-Tajhis, Iran) was used to cDNA synthesis process. The process of reverse transcription in all samples was done at 25 °C (10 min) and 42 °C (60 min), respectively.

*Real Time PCR Studies*

For each sample, the final volume of quantitative Real-time PCR reactions were provided in 20 μL, which contain 1 μL cDNA, 0.5 μL of each forward and reverse primers, and 10 μL RealQ Plus 2xMaster Mix Green, High ROXTM (Ampliqon Inc., Odense, Denmark) which performed in duplicates. The enzyme activation step was done at 95 °C/20 min and followed by 40 cycles at 95 °C/20 s and 58 °C/15 s. At the end of each annealing/extension cycle, the ultimate results were obtained. When the amplification cycles are over, a slow increase in temperature from 60 to 95 °C was used using the StepOne™ instrument (Applied Biosystems, USA) to achieve the melting temperature analysis. The StepOne™ Software v2.2.2 was used to analyze data, and the relative expression of genes were assessed through the ΔΔCT method and REST®2009 bioinformatics software. 

*Western Blot and Protein Expression Studies*

The samples were homogenized in RIPA buffer (Cyto matin gene, Iran) with a protease inhibitor cocktail (Sigma, USA) and centrifuged (15000 rpm, 10 min, 4 °C). The supernatant was collected, and protein content was assessed by the Lowry method. Proteins were separated via sodium dodecyl sulfate-polyacrylamide gel electrophoresis (Bio-Rad, USA) through 4–20% gradient polyacrylamide gels containing 0.1% sodium dodecyl sulfate for about 2 h at 95V. After electrophoresis, the proteins were transferred to polyvinylidene fluoride membrane (PVDF) (Bio-Rad, USA) for 80 min at 80 V. Nonspecific sites have blocked overnight at 4 °C in TBS containing Tween and 5% nonfat milk (Sigma, USA). Membranes were then incubated 2h with primary antibodies directed against Col-I and OCN at room temperature. The protein abundance of GAPDH (which served as a loading control to normalize protein loading and transfer) were determined in all samples. Following incubation with primary antibodies, membranes were washed extensively with PBS-Tween and then incubated with appropriate secondary antibodies for 1 h at RT. After washing, membranes were developed using DAB (3, 3’-diaminobenzidine) substrate, and images of the membrane bands were captured and analyzed using the ImageJ software.

*Statistical Analysis*

The statistical analysis of this study was calculated via SPSS statistical software. To evaluate differences between the results of all electrospun scaffolds and control group, Tukey’s test was applied. Also, the *p*-value of less than or equal to 0.05 were interpreted as being noteworthy. The achieved data was demonstrated in curves as mean ± standard error.

## Results

*Characterization of PAN-based Electrospun Nanofibers*

**Figure 1:**
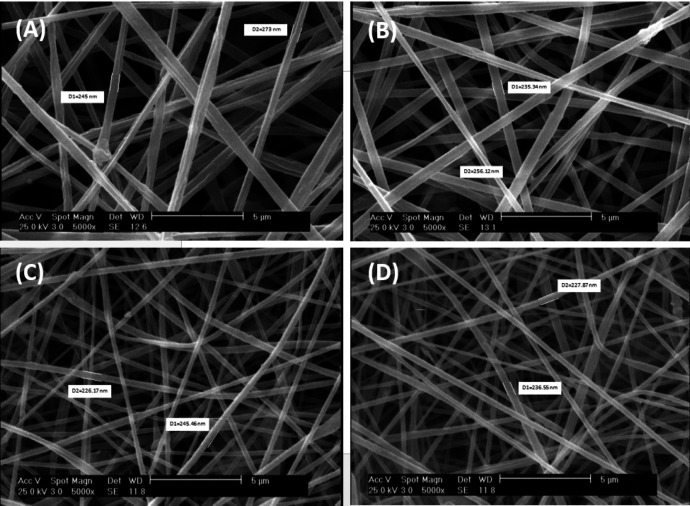
SEM homogenously micrograph of (A) PAN, (B) Clay-PAN 15%, (C) Clay-PAN 20%, (D) Clay-PAN 25% nanofiber electrospun scaffolds with 5 µm scale bar

The reticular, random, and homogenous nanofibers of scaffolds was demonstrated by SEM images (Figure 1). The average diameter of Clay-PAN15%, Clay-PAN20% and Clay-PAN25% and PAN scaffolds were 245 ± 10 nm (SD), 235 ± 10 nm (SD), 232 ± 10 nm (SD), and 259 ± 14 nm (SD) respectively (Figure S1, in supplementary file). SEM images demonstrated the effective dispersion of nanoclay with no agglomerates. So, the addition of nanoclay decrease diameter of nanofibers which can prepare appropriate condition for cells.

**Figure 2:**
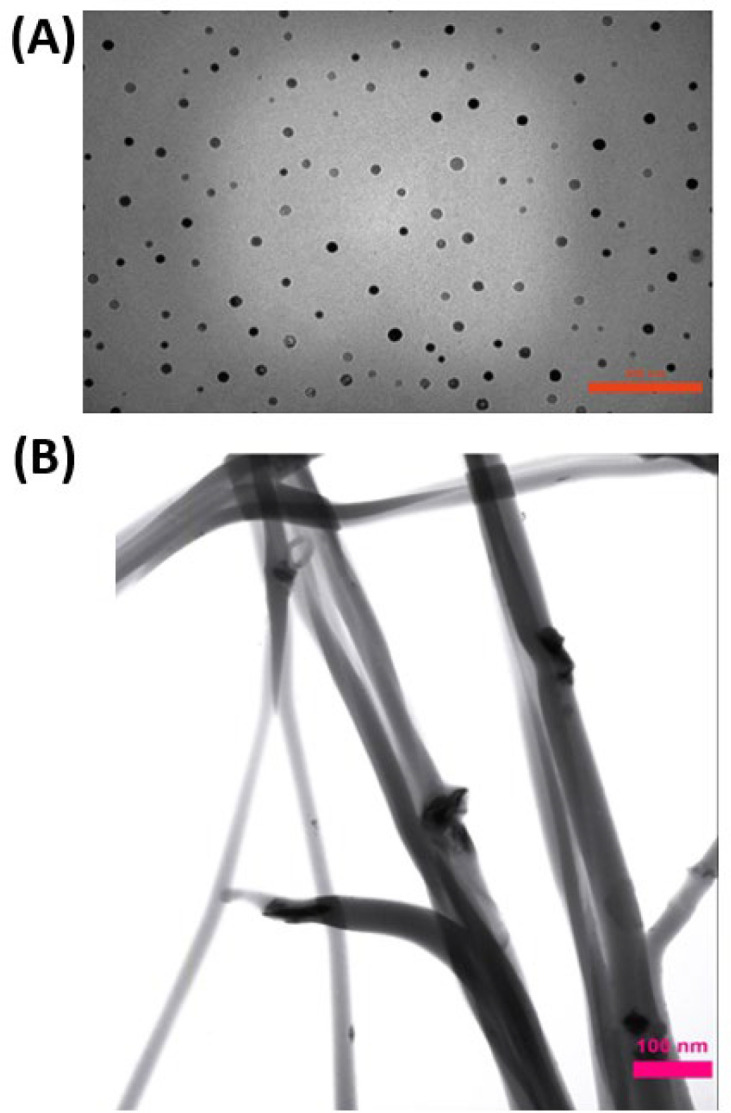
TEM images of (A) Clay nanoparticles, (B) Clay-PAN 25% scaffold with 100 nm scale bar

Clay dispersion of the electrospun composite fibers also was analyzed by TEM images. Images (Figure 2) showed no particle aggregation at 25% clay concentrations. Moreover, images demonstrate that nanoclay was embedded and well dispersed in the nanofiber matrix.

**Figure 3:**
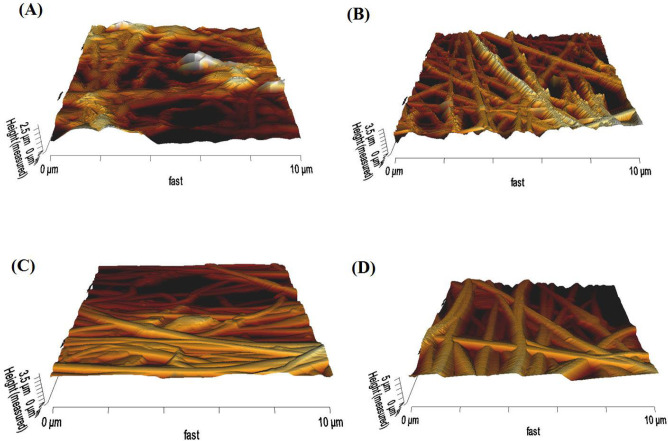
AFM 3D images of (A) PAN, (B) Clay-PAN 15%, (C) Clay-PAN 20%, (D) Clay-PAN 25% nanofiber electrospun scaffolds

The 3D roughness profile of scaffolds was measured using AFM (Figure 3), and the results were converted to roughness values (Ra) (Figure S2, in supplementary file) through JPK data processing instruments. The average roughness of Clay-PAN15%, Clay-PAN20% and Clay-PAN25% and PAN scaffolds was 398.3, 407.5, 630.4 and 253 nm, respectively. The roughness values of Clay-PAN25% was more than the other scaffolds which can prepare more suitable topographic spaces for attachment of cells. In fact, nanoclay with creation of nano scale topography on the surface of scaffolds can influence on the fate of MSCs.

FTIR results recognized the structures, molecular components and functional groups of scaffolds (Figure S3). In all PAN-based scaffolds, the bands at 3000–4000 cm^−1^ revealed OH vibration, and the absorption area in 2244 cm^−1^ was indicated the nitrile bonds. The typical peaks of Na^+^-MMT in Clay-PAN Nanocomposite (CPN) scaffolds, detected at 3622 and 1045 cm^−1^, are recognized as the OH stretching of the lattice water, Si–O and Al–O stretching bond. Moreover, the peaks of PAN are detected at 1662, 1452, 1361, and 1253 cm^−1,^ matching to quinoid ring structure and benzenoid ring structures (24, 25). 

**Figure 4:**
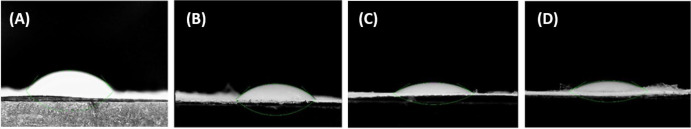
Contact angel micrograph of (A) PAN, (B) Clay-PAN 15%, (C) Clay-PAN 20%, (D) Clay-PAN 25% nanofiber electrospun scaffolds

Contact angle test demonstrated the surface wettability of the scaffolds. Figure 4 demonstrated that all scaffolds were classified as hydrophilic surfaces, which was a positive point for cell attachment. The contact angle of Clay-PAN15%, Clay-PAN20% and Clay-PAN25% and PAN nanofibers of scaffolds was 43.154˚, 31.007˚, 30.226˚ and 51.169˚, respectively. So, the hydrophilic properties of Clay-PAN25% scaffold were richer than other PAN-based scaffolds, making it suitable for cell attachment.

The tensile test evaluated the mechanical properties of scaffolds. Table 2 demonstrated the results of the tensile examination of scaffolds. The Clay-PAN25% demonstrated an obvious increase in tensile strength and modulus compared to the Clay-PAN15%, Clay-PAN20% and PAN scaffold. It was maybe because of the improvement of the scaffold crystallinity and the development in the mobility of polymeric chains caused by nanoclay. 

The zeta potential is related to the superficial charges of different scaffolds. Nanoparticles with the cationic surface are more cytotoxic in general, and they are prone to induce lysosomal damages, so the nanoparticles with the anionic surface are preferred (26). So, the addition of nanoclay to the solution of scaffolds caused to generate anionic surface charges (Figure S4), and Clay-PAN25% was the best scaffold due to the highest surface anionic charge. 

**Figure 5:**
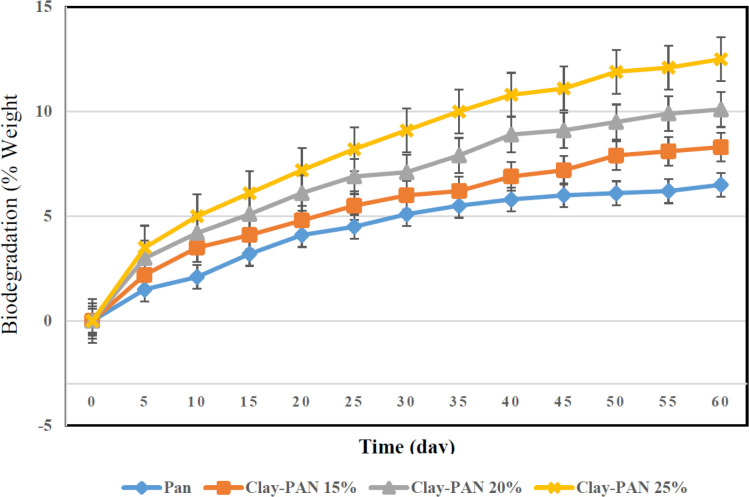
The degradation behaviors of the scaffolds after PBS immersion

The biodegradation activities of the scaffolds were considered by weight loss measurement in PBS at 37 °C. The weight loss of the scaffolds with 0–13 wt% of clay content over a 60 day period was obtained through gravimetric analysis. The weight loss of the scaffolds increased with increasing clay content of materials (Figure 5).

*Mesenchymal Stem Cell Characterization Analysis*

AD-MSCs (passage2) were characterized by surface markers via flow cytometric analysis. According to flow cytometry results (Figure S5) the white curve indicated the isotype control, and the colored curve indicated the targeted CD markers. AD-MSCs were positive for CD44, DC73, CD90, CD105 and negative for CD45, CD34.

*Biocompatibility Study Analysis*

**Figure 6:**
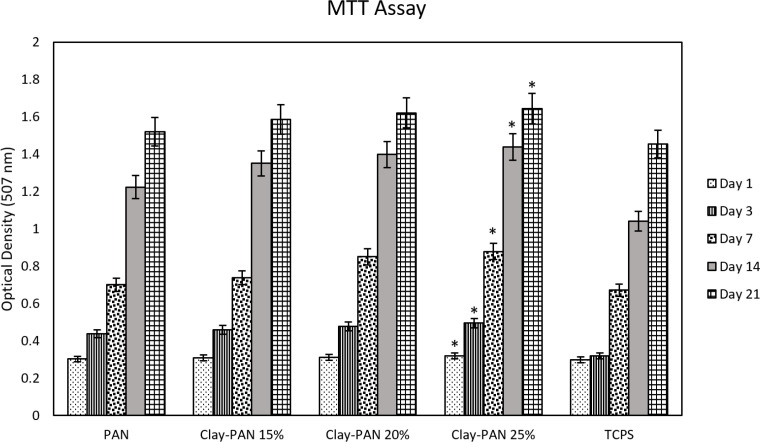
MTT results of nanofiber electrospun scaffolds. The * sign indicates a significant difference (*p*-value ≤ 0.05) in Clay-PAN 25% group

To investigate the viability and cyto-compatibility of scaffolds, the MTT method as a colorimetric assay was used at 570 nm. The optical density of samples was assessed, and their absorbance values (mean and standard deviation) were measured after 1, 3, 7, 14, and 21 days (Figure 6). The significant differences between Clay-PAN15%, Clay-PAN20%, and Clay-PAN25% scaffolds with control groups (PAN scaffold and TCPS) were observed (*p *< 0.05). The biocompatibility of Clay-PAN scaffolds was more than PAN, PAN-DM (PAN with differentiated medium), and TCPs groups.

*Cell Adhesion Studies Analysis*

**Figure 7:**
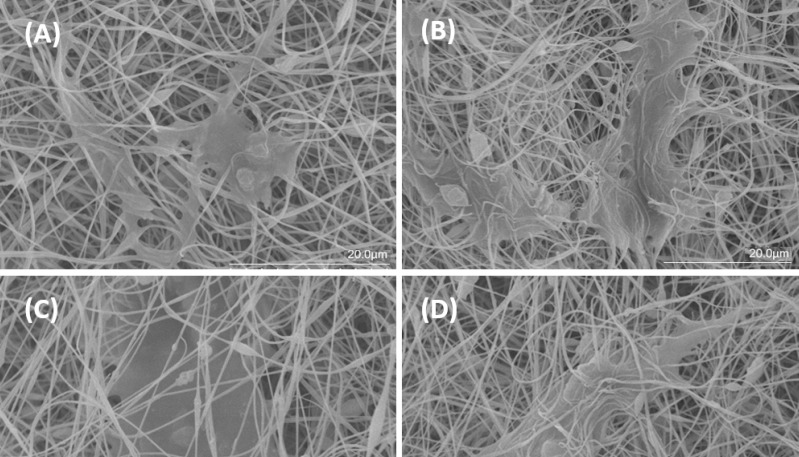
SEM surface morphology of (A) PAN, (B) Clay-PAN 15%, (C) Clay-PAN 20%, (D) Clay-PAN 25% nanofiber electrospun scaffolds after cell seeding at day 21 with 20 µm scale bar

AD-MSCs attachment to scaffolds was evaluated through SEM and DAPI test. SEM images (Figure 7) demonstrated that AD-MSCs adhered well to Clay-PAN15%, Clay-PAN20%, Clay-PAN25%, and PAN scaffolds and surface of the scaffolds created a suitable microenvironment for cell–cell and cell–matrix connection after 21 days. SEM images confirm the results of the biocompatibility test. 

DAPI staining was performed on days 14 and 21. Images (Figure S6) were taken through fluorescence microscopy. It represented the adhesion of MSCs with a healthy nucleus to all scaffolds, and it was shown in blue color with a dark field. The assessed cell density in Clay-PAN 15%, Clay-PAN20%, Clay-PAN25% and PAN scaffolds was 5, 18, 30 and 15 cell/100 µm^2^ in days 14 and 10, 20, 36 30 cell/100 µm^2^ in day 21, respectively. 

*Masson’s Trichrome and ALP Activity Studies for Osteogenic Analysis*

Masson’s trichrome staining represented the collagen deposition rate in 14 and 21 days with blue-stained tissue. The proportions of collagen in the Clay-PAN15%, Clay-PAN20% and Clay-PAN25% scaffolds were (38.10 ± 0.5) %, (41.39 ± 0.5) %, (48.61 ± 0.5) % in day 14 and (40.14 ± 0.5) %, (55.39 ± 0.5) %, (69.61 ± 0.5) % in day 21, and those in the PAN scaffold as control group were (31.19 ± 0.5) % and (21.52 ± 0.5) %, respectively. In bone tissue, collagen increased gradually with bone formation, and the discrepancy between all samples showed that collagen production by AD-MSCs on Clay-PAN25% scaffold was 1.2% to 1.4% more than Clay-PAN 15% and Clay-PAN 20% scaffolds, and 1.5% to 3.2% more than the PAN scaffold, which indicated that bone formation in the Clay-PAN25% scaffold was significantly more than other scaffolds (p < 0.05, Figure S7).

**Figure 8:**
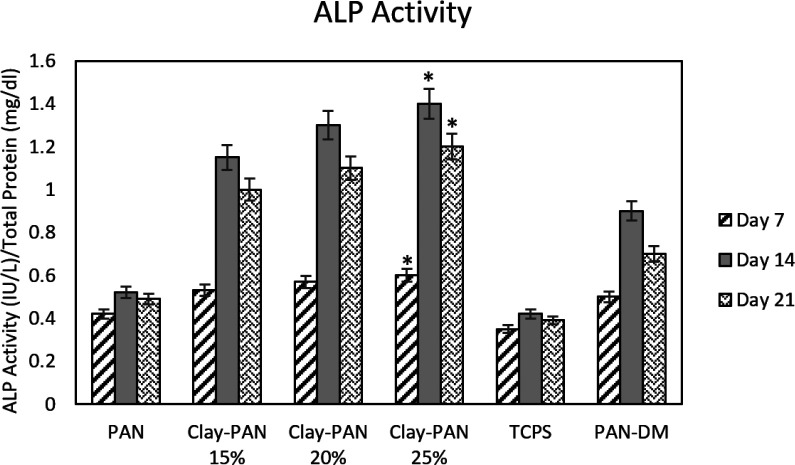
ALP results of nanofiber electrospun scaffolds. The * sign indicates a significant difference (*p*-value ≤ 0.05) in Clay-PAN 25% group

ALP activity was assessed on days 7, 14, and 21. ALP results showed a higher activity pattern at day 14, and the highest ALP activity was observed in the Clay-PAN25% scaffold compared with other groups. There were significant differences between Clay-PAN15%, Clay-PAN20%, and Clay-PAN25% scaffolds with control groups (*p *< 0.05, Figure 8).

*Real Time PCR Results Analysis*

**Figure 9:**
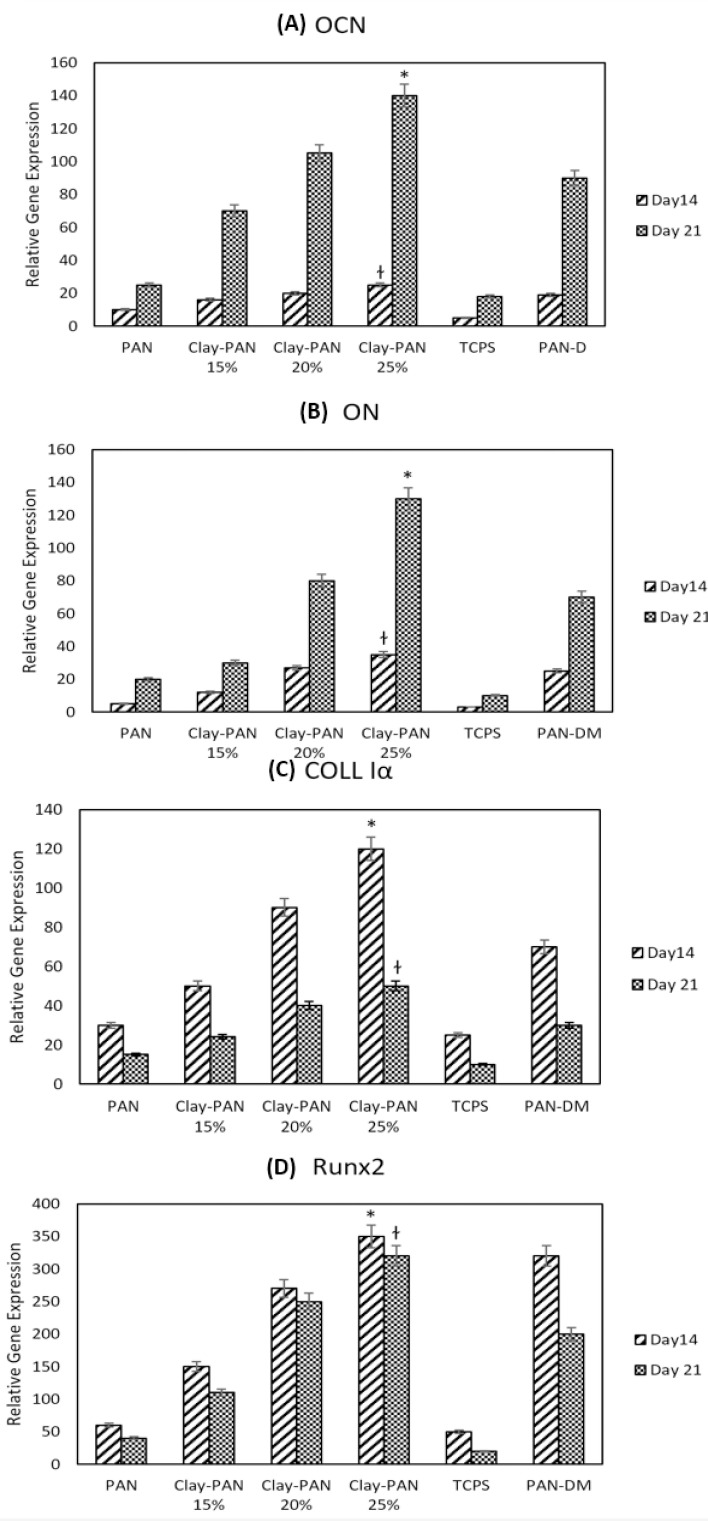
Normalized relative expression of (A) Osteocalcin (OCN), (B) Osteonectin (ON), (C) Collagen type I *alpha* (Col Iα) (D) and Runt related transcription factor 2 (RUNX2) genes of AD-MSCs on PAN, Clay-PAN 15%, Clay-PAN 20%, Clay-PAN 25%, TCPS and PAN-DM (PAN with differentiated medium) groups at days 14 and 21. The *, † sign indicate a significant difference (*p*-value ≤ 0.05) in Clay-PAN 25% group

RUNX2, Colα, OCN, and ON are the most important osteogenic differentiation genes. Relative expression of target genes was evaluated at 14 and 21 days to indicate the osteogenic differentiation potential of AD-MSCs on scaffolds. Real-time PCR results indicated that the expression of OCN and ON, as of late genes, was higher at day 21, and the expression of Runx2 and Coll1α, as early genes, was higher at day 14 in all samples. This proved the occurrence of the bone differentiation process in all groups. Also, the highest expression of all mentioned genes belongs to the Clay-PAN25% group, and the lowest expression was observed in the TCPs group on days 14 and 21. This indicated the effect of nanoclay on osteogenic gene expression and proved that the higher concentrations of nanoclay help to more genes expression. In addition, gene expression in Clay-PAN25% (with DMEM medium) was higher than PAN-DM (with differentiated medium) group in both 14 and 21 days, which indicates the positive effect of nanoclay presence in the osteogenic differentiation process. The results of all gene expressions were normalized with the GAPDH gene (*p *< 0.05, Figure 9).

*Western Blot Analysis*

**Figure 10:**
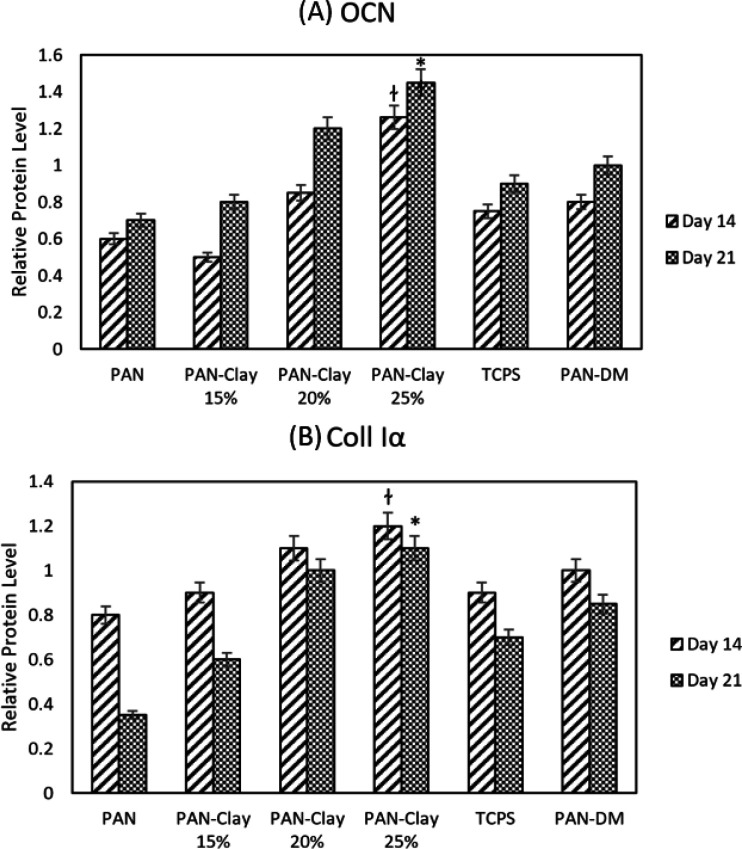
The western blot diagram of AD-MSCs on PAN, Clay-PAN 15%, Clay-PAN 20%, Clay-PAN 25%, TCPS and PAN-DM (PAN with Differentiated medium) groups for (A) Osteocalcin(OCN) and (B) Collagen type I proteins at days 14 and 21. The *, † sign indicate a significant difference (*p*-value ≤ 0.05) in Clay-PAN 25% group

The protein concentration measurements was performed by Lowry method. Total proteins are obtained from cell lysates. Protein quantification was performed in triplicates which followed by ImageJ and ANOVA program analysis. OCN and Col1 were selected to assess the protein quantification by western blotting on days 14 and 21. The measurements of protein concentration demonstrated that the expression of OCN was higher at day 21 and the expression of Coll1α was higher at day 14 in all samples which proved the occurrence of osteogenesis in all groups. Also the highest expression of OCN and Coll1α was belong to the Clay-PAN25% group. Both protein expression in Clay-PAN20% and Clay-PAN25% groups was higher than PAN-DM group in 14 and 21 days, which indicates the positive effect of nanoclay in compare with osteogenic differentiated medium (Figures 10 and S8). The difference between the control groups and the other groups was statistically significant (*p* < 0.05). GAPDH protein was considered as a control. The expression of GAPDH protein was positive in all samples, which indicates the accuracy of the test. 

## Discussion

Trauma, congenital defects, and tumor resection can cause severe damage to bone tissues. Efficient treatment of bone defects is one of the major challenges in medicine (27). Difficulties of existing treatment procedures led to the search for alternative methods. The tissue engineering (TE) approach is a collection of engineered materials, biologically active molecules, chemical or physical parameters, and stem cells (28). Scaffolds are three-dimensional substrates involved in the binding of cells and play a significant role in tissue repair and regeneration by preparing a suitable platform, mimicking ECM conditions, as well as providing various factors associated with proliferation, differentiation, and migration of cells (29). Polymeric scaffolds are gradually reducing the need for bone grafts^5^. Polyacrylonitrile (PAN) was applied in this study because of its good mechanical resistance properties. Since the scaffold used in bone TE must be physically constant in the implanted site, designing its structure is crucial (30). Nowadays, various methods are applied for the fabrication of nanomaterial scaffolds (31). The electrospinning technique is one of the most promising methods used in bone TE due to its mimicking properties of ECM and its speed of operation, easiness, and construction of various nanofibers (24). Electrospinning has been widely used to create nanofibers with a high and tunable porosity, high surface area, and diameters similar to natural ECM (32). Until now, different types of scaffolds have been fabricated by electrospinning and exploited for TE (33). Wong* et al. *(2014) investigated the role of electrospun PCL scaffold in the differentiation of MC3T3-E1 preosteoblasts. The results showed that these nanofibers increase cell adhesion, proliferation, and differentiation of MC3T3-E1 cells (34). In the present study, four types of nanofiber scaffolds were synthesized via the electrospinning method to evaluate cell differentiation. In addition, because of the higher melting point and greater carbon yield, PAN-based nanofibers widely are applied for producing high-performance carbon nanofibers via a heat treatment process of electrospun nanofibers (35). Mohamadali *et al.* (2017) fabricated biocompatible electrospun PANi/PAN scaffolds for studying the proliferation and differentiation of MSCs to muscle-like cells, which showed enhanced proliferation and differentiation to achieve muscle-like cells (24). So, in the present study, four electrospun PAN-based nanofiber scaffolds were synthesized and used in cell culture and bone differentiation. Obviously, there are different cell sources for cell differentiation studies that were selected based on the target of research. For instance, Shafiei *et al.* (2016) applied AD-MSCs as a cell source in order to differentiate AD-MSCs on fabricated electrospun hydroxide (LDH)/poly(ε-caprolactone) (PCL) nanocomposite. Their results showed the excellent potential of their scaffold to AD-MSCs differentiation and its application in soft TE (36). Similar to this study, AD-MSCs were determined as the cell source of the present study. In addition to the cellular source, the surface properties of scaffolds are important in the fate of MSCs. Surface characteristics of scaffolds, including topography, stiffness, surface free energy, surface roughness, chemical functionalities, surface charge, and wettability, are important parameters that play a key role in cell interactions with the scaffolds, modulating the behavior of cells, as well as inducing osteogenic differentiation of stem cells (37, 38). Various approaches have been applied in order to modify the surface properties of scaffolds (39). One of these approaches is the use of nanoparticles which, due to their unique properties, have been applied in a wide variety of TE fields to improved biological and mechanical performances and regulated cell processes (9, 40 and 41). Various types of nanomaterials, including Ag nanoparticles, MgO nanoparticles (42), hydroxyapatite, TiO2 nanoparticles, carbon nanotubes, and graphene oxide, have been applied nowadays to reinforce surface properties (such as topography, charge, and roughness) of electrospun scaffolds (40, 43). Several studies verified the crucial role of surface topography in regulating cellular activities such as adhesion, proliferation and differentiation on 2D surfaces (44, 45). The resuresearch have shown thatd shown nano/micro scale topography can influence cell activitmodifyingtion of cytoskeleton arrangements (46). Different methods including polymer phase separation, photo/electronbeam lithography and electrospinning can prepare nano-scale topographies (47). Studies suggested that a stiff interface with a micro/nano scale surface topography that mimics collagenous bone would support osteogenic differentiation of the cells (33). In fact, topography of the scaffold can influence on focal adhesions (FA) formation, which leads to changes in morphology and shape of cells, and eventually affecting cells proliferation and differentiation into specific cell lineage by different signaling pathways activation (44, 48). Nokhaste *et al*., found that bioactive glass nanoparticles could significantly alter the surface chemistry and topography of the PLGA/collagen scaffolds and lead to better proliferation of fibroblast cells (49). In the present study, nanoclay were used in the structure of scaffolds to make changes in topography and surface properties of PAN scaffolds and to induce osteogenesis in stem cells. Indeed, the presence of nanoclay reduced the diameter of the nanofibers, increased the wettability and surface roughness as well as surface charges of PAN-based scaffolds. These changes may be due to its specific chemical structure of nanoclay such as the presence of negative silica groups on the outer surfaces. Studies have been performed to investigate the effect of surface charge in the cell adhesion mechanism (50, 51). Olthof *et al*. indicated the different effects of neutral, negative and positive surface charges of the scaffolds on the bone formation process (52). Also, according to the previous studies, enhanced surface roughness can improve the biocompatibility of scaffold materials (33) and boost the initial cell adhesion (53). Tang *et al*, demonstrated the effect of silica nanoparticles on the fiber surface of a polycaprolactone fibrous scaffold in improvement of surface roughness and fiber wettability of scaffold (54). In the present study, the presence of nanoclay in PAN-based scaffolds had a positive effect on the surface charges and surface roughness of the scaffolds. Nanoclay caused more negative surface charge and enhanced the surface roughness of scaffolds. On the other hands, nanoparticles have been found that can induce the differentiation of cells (55). Karimi *et al*., electrospun PLLA nanofibrous scaffold with Baghdadite nanparticles and the potential of scaffolds for regeneration of bone was investigated by using AD-MSCs. PLLA-Baghdadite indicated the capability to induce expression of osteogenesis-related genes such as RUNX2, ALP and OCN (56). In the present study, nanoclay in different concentration was applied in the material of PAN-based scaffolds to assess the changes that occur in the surface properties of scaffolds and the osteogenic differentiation potential of AD-MSCs. We found that the presence of 25% of nanoclay can affect positively on surface charges and roughness of scaffolds and can induce osteogenesis differentiation in AD-MSCs through increasing the ALP activity, proportions of collagen and enhancing osteogenic genes and protein expression. Studies showed that nanoclay are a kind of nanomaterial with wide applications in TE (57). The negative silanol groups present on the outer surface of clay minerals were found to serve as one of the major sites of electrostatic interaction with cationic groups on positively charged polymers. While, electrostatic interactions on the positive rims of clay minerals, ligand exchange, van der Waals interactions as well as cation bridging on the negative clay surfaces may absorb negatively charged polymers (10). Various studies have focused on nanoclay effects on bone differentiation and the cellular functions of skeletal populations (10). Villaça *et al*. prepared clay mineral-polymer membranes based on chitosan, sodium alendronate (ALN) and Sodium montmorillonite (Na-Mt). Membranes obtained from nanocomposites indicated to have the ability to induce the proliferation and differentiation of human osteoblast-like cell line (Saos-2 cells) (58). In our previous study, we loaded clay and graphene nanoparticles into PAN-based scaffolds in order to evaluate their effects in promoting bone differentiation of AD-MSCs. Obtained results indicated both of nanoparticles have positive effects on osteogenic differentiation (22), however, the effective mechanisms of differentiation progression are still unclear (10). In present study, the presence of nanoclay in the material of scaffolds is useful to induce osteogenic differentiation in MSCs. First, we characterized fabricated scaffolds. SEM images showed the homogenous, random and reticular nanofibers and the average diameter of scaffolds were measured. It became clear that the addition of nanoclay decreases diameter of electrospun fibers and it approved the effective dispersion of nanoclay with no agglomerates in nanofibers. Clay dispersion of the fibers were analyzed by TEM images which showed well clay dispersed without particle aggregation at Clay-PAN25% nanofibers. Based on AFM results, the 3D profile of the all scaffolds demonstrated the average roughness of scaffolds which approved the addition of nanoclay could increase surface roughness of nanofibers. Moreover, the roughness values of Clay-PAN25% was more than the other scaffolds which can prepare more suitable topographic spaces for attachment of stem cells. Based on FTIR results, the specific peaks of scaffolds were observed. Also, the wettability of the material surface was measured by contact angle which demonstrated the hydrophilic properties of the scaffolds which was more in Clay-PAN25% and made it as a suitable area for attachment of cells. The mechanical properties of scaffolds were assessed by tensile test which demonstrated an increase in tensile strength, modulus and elongation of Clay-PAN25% compared to the others. The superficial charges of all scaffolds were measured through zeta potential test which approved the Clay-PAN25% scaffold has the highest surface anionic charges. Also, the biodegradation activities of the scaffolds showed the weight loss of scaffolds were increased with increasing clay content of materials. In next step, MSCs was isolated from human adipose tissues and characterized through flowcytometry analysis. Then the biocompatibility of scaffolds was approved by MTT assay. Also, the attachment of cells was demonstrated by DAPI staining and SEM images. In final step, the osteogenesis potential of AD-MSCs have been assessed. In this way, mansson’s trichrome staining was approved the superiority of Clay-PAN25% scaffold for osteogenic differentiation of AD-MSCs at days 14 and 21. Based on previous studies, collagen progressively increased with bone formation which approved in present study too. Based on ALP results, the highest enzyme concentration was observed at day 14 which is anticipated that the maximum amount of ALP activity is in the mid-differentiation. ALP activity in Clay-PAN25% scaffold on days 14 and 21 was higher than other groups. ALP activity of Clay-PAN25% scaffold with DMEM medium was higher than PAN scaffold with differentiated medium (PAN-DM) which demonstrated the effect of nanoclay in osteogenic differentiation. On the other hands, osteogenic genes and protein expression data confirmed the superiority of Clay-PAN25% scaffold for bone differentiation due to the highest expression of osteogenic genes and proteins. Based on this study, relative expression of RUNX2, Colα, OCN and ON genes was evaluated in days 14 and 21. The expression of late osteogenic genes was higher and expression of early osteogenic genes was lower at day 21. Also, the expression of all mentioned genes in Clay-PAN15%, Clay-PAN20% and Clay-PAN25% groups was significantly higher than that of TCPS group on 14 and 21 days which could be related to the presence of nanoclay in the material of these scaffolds. These data were confirmed more by the results of western blot assay. Based on acquired results, the OCN and Col1 protein had expression in all groups on days 14 and 21 which indicated the osteogenesis differentiation process and the Clay-PAN25% had the highest expression of OCN and Col1 protein on day 21 which embossed the Clay-PAN25% nanofibers as a suitable scaffold in bone regeneration studies. Therefore, the current study demonstrated that the presence of 25% of nanoclay in PAN-based scaffolds could guide the MSCs to follow the traces of bone differentiation process. Indeed, the existence of nanoclay in the PAN-based scaffolds can cause cell differentiation without the presence of osteogenic growth factors. In TE, finding a suitable alternative to the differentiation medium is very ideal because with the help of nanomaterials, the required amount of chemical factors can be reduced or removed from the cell culture medium. In this study, bone differentiation was reported via Clay-PAN scaffolds without osteogenic growth factors, which led to cost reduction and economic efficiency. As a result, clay nanoparticle can influence on MSCs behaviors such as adhesion, alignment, proliferation, migration and differentiation besides the effect on the surface properties of scaffolds such as topography, roughness, surface charge and wettability. However, the supplementary studies in *in-vivo *condition are still required to demonstrate the long-term fate of the nanoclay before clinical applications in future.

**Table 1:** The sequences of specific primers for osteogenic differentiation genes

**Gene names**	**Primer sequences**
h-COL1A1-F	TTGTGGATGGGGACTTGTGA
h-COL1A1-R	AGAGGCAGGTGGAGAGAGG
h-ON-F	TAGAGGCTAAGTGGTGGGAGA
h-ON-R	TGAAAGGTAAAGGAGGAAATGGT
h-OCN-F	CCAAGGAGGGAGGTGTGTGAG
h-OCN-R	AAGGGGAAGAGGAAAGAAGGGTG
hGAP-F	GCA GGG ATG ATG TTC TGG
hGAP-R	CTT TGG TAT CGT GGA AGG AC
h-RUNX2-F	TCTTAGAACAAATTCTGCCCTTT
h-RUNX2-R	TGCTTTGGTCTTGAAATCACA

**Table 2:** Tensile properties of the the electrospun nanofibrous scaffolds. Clay-PAN 25% demonstrated an obvious increase in tensile strength and modulus

**Sample**	**Max Tensile Strain (%)**	**Max Tensile strength (Mpa)**	**Tensile modulus**
Clay-PAN 25%	0.71 ± 0.1	2.11 ± 0.4	2.97 ± 0.2
Clay-PAN 20%	2.72 ± 0.5	0.22 ± 0.1	0.08 ± 0.2
Clay-PAN 15%	1.50 ± 0.3	0.25 ± 0.1	0.16 ± 0.3
PAN	0.64 ± 0.1	0.51 ± 0.2	O.79 ± 0.2

## Conclusion

This study demonstrated *in-vitro* bone differentiation of AD-MSCs on PAN-based nanofiber electrospun scaffolds with different concentrations of nanoclay for the first time. It approved that nanoclay has an effective consequence on MSCs behaviors such as adhesion and differentiation and affects the surface properties of scaffolds such as topography, surface charge, and roughness. Briefly, in this study, the structure of scaffolds was assessed. After cell isolation, cell characterization, cell attachment, and scaffolds biocompatibility were determined. Finally, AD-MSCs osteogenic differentiation was confirmed. As a result of this study, the Clay-PAN scaffold was introduced as a suitable support for attachment, proliferation, and differentiation of MSCs. Clay-PAN25% nanofiber electrospun scaffold demonstrated the best results in comparison with PAN and other clay-PAN scaffolds at the same condition. An interesting result of this study was bone differentiation via nanoclay presence without osteogenic growth factors. So, the use of nanoclay in the construction of scaffolds has an important effect on the topography of scaffolds and, eventually the fate of MSCs. So, Clay-PAN scaffolds can mimic the ECM of bone tissue due to the presence of clay mineral nanoparticles, making it a promising candidate for bone tissue regeneration studies in the future.

## Funding sources

The corresponding research was done using the grant (ID: 12622) of Shahid Beheshti University of Medical Sciences.

## Ethical statement

This study is under the support of Shahid Beheshti University’s Medical Research Ethics Committee (IR.SBMU.REC.1397.02).

## Competing interests

There are no competing interests to declare.

## Authors’ contribution

All authors equally contributed to this study.
